# Multiple modality biomarker prediction of cognitive impairment in prospectively followed de novo Parkinson disease

**DOI:** 10.1371/journal.pone.0175674

**Published:** 2017-05-17

**Authors:** Chelsea Caspell-Garcia, Tanya Simuni, Duygu Tosun-Turgut, I-Wei Wu, Yu Zhang, Mike Nalls, Andrew Singleton, Leslie A. Shaw, Ju-Hee Kang, John Q. Trojanowski, Andrew Siderowf, Christopher Coffey, Shirley Lasch, Dag Aarsland, David Burn, Lana M. Chahine, Alberto J. Espay, Eric D. Foster, Keith A. Hawkins, Irene Litvan, Irene Richard, Daniel Weintraub, the Parkinson’s Progression Markers Initiative (PPMI)

**Affiliations:** 1 Department of Biostatistics, College of Public Health, University of Iowa, Iowa City, IA, United States of America; 2 Feinberg School of Medicine, Northwestern University, Chicago, IL, United States of America; 3 University of California, San Francisco, San Francisco, CA, United States of America; 4 Laboratory of Neurogenetics, National Institute on Aging, National Institutes of Health, Bethesda, MD, United States of America; 5 Department of Pathology and Laboratory Medicine, Perelman School of Medicine at the University of Pennsylvania, Philadelphia, PA, United States of America; 6 Department of Pharmacology & Clinical Pharmacology, Inha University School of Medicine, Incheon, Republic of Korea; 7 Avid Radiopharmaceuticals, Philadelphia, PA, United States of America; 8 Institute for Neurodegenerative Disorders (IND) and Molecular NeuroImaging, LLC (MNI), New Haven CT, United States of America; 9 Department of Old Age Psychiatry, Institute of Psychiatry, Psychology & Neuroscience, King’s College London, London, England; 10 Centre for Age-Related Medicine, Stavanger University Hospital, Stavanger, Norway; 11 Institute for Ageing and Health, Newcastle University, Newcastle, England; 12 Department of Neurology, Perelman School of Medicine at the University of Pennsylvania, Philadelphia, PA, United States of America; 13 Department of Neurology, University of Cincinnati Academic Health Center, Cincinnati, OH, United States of America; 14 Department of Psychiatry, Yale School of Medicine, New Haven, CT, United States of America; 15 UCSD Movement Disorder Center, Department of Neurosciences, University of California San Diego, San Diego, CA, United States of America; 16 Departments of Neurology and Psychiatry, University of Rochester School of Medicine and Dentistry, Rochester, NY, United States of America; 17 Department of Psychiatry, Perelman School of Medicine at the University of Pennsylvania, Philadelphia, PA, United States of America; 18 Parkinson’s Disease Research, Education and Clinical Center (PADRECC and MIRECC), Philadelphia Veterans Affairs Medical Center, Philadelphia, PA, United States of America; 19 Mental Illness Research, Education and Clinical Center (MIRECC), Philadelphia Veterans Affairs Medical Center, Philadelphia, PA, United States of America; Nathan S Kline Institute, UNITED STATES

## Abstract

**Objectives:**

To assess the neurobiological substrate of initial cognitive decline in Parkinson’s disease (PD) to inform patient management, clinical trial design, and development of treatments.

**Methods:**

We longitudinally assessed, up to 3 years, 423 newly diagnosed patients with idiopathic PD, untreated at baseline, from 33 international movement disorder centers. Study outcomes were four determinations of cognitive impairment or decline, and biomarker predictors were baseline dopamine transporter (DAT) single photon emission computed tomography (SPECT) scan, structural magnetic resonance imaging (MRI; volume and thickness), diffusion tensor imaging (mean diffusivity and fractional anisotropy), cerebrospinal fluid (CSF; amyloid beta [Aβ], tau and alpha synuclein), and 11 single nucleotide polymorphisms (SNPs) previously associated with PD cognition. Additionally, longitudinal structural MRI and DAT scan data were included. Univariate analyses were run initially, with false discovery rate = 0.2, to select biomarker variables for inclusion in multivariable longitudinal mixed-effect models.

**Results:**

By year 3, cognitive impairment was diagnosed in 15–38% participants depending on the criteria applied. Biomarkers, some longitudinal, predicting cognitive impairment in multivariable models were: (1) dopamine deficiency (decreased caudate and putamen DAT availability); (2) diffuse, cortical decreased brain volume or thickness (frontal, temporal, parietal, and occipital lobe regions); (3) co-morbid Alzheimer’s disease Aβ amyloid pathology (lower CSF Aβ 1–42); and (4) genes (*COMT* val/val and *BDNF* val/val genotypes).

**Conclusions:**

Cognitive impairment in PD increases in frequency 50–200% in the first several years of disease, and is independently predicted by biomarker changes related to nigrostriatal or cortical dopaminergic deficits, global atrophy due to possible widespread effects of neurodegenerative disease, co-morbid Alzheimer’s disease plaque pathology, and genetic factors.

## Introduction

In Parkinson disease (PD) cognitive impairment can occur in a range of cognitive domains[1], dementia (PDD) affects up to 80% of patients long-term[2], mild cognitive impairment (PD-MCI) occurs in 25–30% of non-demented patients[1] and is a risk factor for dementia[3], and cognitive deficits are present in some patients at the time of diagnosis[4].

A range of demographic and clinical correlates or potential risk factors for cognitive decline have been identified, including increasing age and duration of PD, male sex, specific motor features (postural instability gait disorder [PIGD] subtype), and a range of non-motor symptoms (e.g., visual hallucinations, apathy, depression, and rapid eye movement (REM) sleep behaviour disorder)[5].

Cortical Lewy body disease (LBD) pathology appears to be the major contributing pathology to cognitive decline in PD[6], but Alzheimer disease (AD)-related changes are also present in a significant percentage of patients[7]. A range of neurotransmitter deficits have been implicated, including in acetylcholine[8], dopamine[9], and norepinephrine systems[10]. Genetic influences have been identified in some studies, including apolipoprotein E4 [*ApoE4*] status[11] and SNPs in brain-derived neurotrophic factor (*BDNF*) val^66^met[12], catechol-O-methyl-transferase (*COMT*) val^158^met[13], and microtubule-associated protein tau (*MAPT*)[14]. Finally, diffuse (primarily medial temporal lobe, parietal lobe and prefrontal cortex) gray matter atrophy and white matter changes have been associated with cognitive decline in PD[15, 16].

The research on the neural substrates of the initial stages of cognitive decline in PD, starting with disease onset, are limited, with previous studies often characterized by single site participation, relatively small sample sizes, cross-sectional design, or a limited biomarker assessment. The Parkinson’s Progression Markers Initiative (PPMI) is an ongoing, prospective, longitudinal, biomarker-rich observational study of disease progression in early PD[17]. The biomarkers obtained in the PPMI study include dopamine transporter (DAT) SPECT imaging, brain structural MRI, CSF and blood biomarkers as well as DNA for genotyping. The goals of these analyses were to evaluate which baseline and longitudinal biomarkers may predict cognitive impairment in early PD.

## Materials and methods

### Participants

Newly diagnosed, untreated PD patients (N = 423) were enrolled in PPMI from June 2010—May 2013 out a cohort of 489 screened patients. At baseline PD participants were required to: (1) have a recent idiopathic PD diagnosis; (3) be untreated for PD; (4) have a dopamine transporter (DAT) deficit on imaging; and (5) not have dementia as determined by the site investigator. The aims and methodology of the study have been published elsewhere[17] and are available at www.ppmi-info.org/study-design. The overall study was approved by the Research Subjects Review Board at the University of Rochester, and the study was approved by the institutional review board at each site, and participants provided written informed consent. Clinical data out to three years post-baseline was utilized. Data was downloaded on September 21, 2015; at the time of data download 38 PD patients had discontinued study participation (9.0% discontinuation).

### Experimental design

#### Cognitive abilities

Cognition was assessed at baseline and annually. Global cognition was assessed with the Montreal Cognitive Assessment (MoCA). In addition, a detailed cognitive battery, as previously described and referenced[18], assessing the following domains was administered: memory (Hopkins Verbal Learning Test-Revised [HLVT-R]); visuospatial function (Benton Judgment of Line Orientation [JOLO]) 15-item (split-half) version; processing speed-attention (Symbol-Digit Modalities Test [SDMT]); and executive function and working memory (Letter-Number Sequencing [LNS] and semantic fluency [animals, vegetables and fruits]). Level II PD-MCI criteria[19] were not applicable given the lack of a separate language assessment or 10 cognitive tests across 5 domains. Published norms for each test were applied.

#### Definitions of cognitive impairment

For the purposes of these analyses cognitive impairment was defined three different ways:

1. The recommended MoCA cut-off for PD of <26 was applied[20]. Additionally, MoCA score was also examined as a continuous variable.
2. Using the detailed cognitive battery, cognitive impairment was defined as at least two test scores >1.5 standard deviations below the standardized mean score, a level of impairment within the recommend range (>1.0–2.0) of standard deviations below the mean to support a PD-MCI diagnosis[19]. Single scores were generated for each test, except for the HVLT-T, for which two scores were used (immediate free recall and recognition discrimination).
3. The site investigator’s clinical diagnosis of cognitive impairment (PD-MCI or PDD) versus no cognitive impairment was made annually. Each site investigator was provided an instruction sheet that outlined how to assess cognitive decline, functional impairment, and general interpretation of cognitive tests to make a diagnosis of PD-MCI[19] or PDD[21]. As previously described [18], the site investigator’s annual determination of cognitive impairment was introduced after some participants already had completed their baseline and year 1 visits (106/423 [25.1%] of available patients had this assessment performed at baseline, and 271/395 [68.6%] at year 1).

Secondary analyses examined only incident cognitive impairment, including only those participants who did not meet one of the three criteria for cognitive impairment at the baseline visit (N = 394).

#### Biomarkers

Details about the biospecimen collection and analysis has been published[17].

1. DAT SPECT imaging (DaTscan^TM^) was obtained at baseline and annually. Ipsilateral (i.e., brain hemisphere on *same* side as predominant motor symptoms) and contralateral (i.e., brain hemisphere on *opposite* side as predominant motor symptoms) caudate and putamen values were used.
2. CSF was obtained at baseline, month 6, year 1, and then annually using collection steps as described [22]. At the time of data download, values were available only for the baseline visit. Reported are levels of alpha synuclein (α-synuclein), total tau, p-tau181, beta-amyloid 1–42 (Aβ42), t-tau:Aβ42 ratio, p-tau181:Aβ42 ratio, and p-tau181:total tau ratio. These CSF biomarkers were measured in centralized laboratories using the xMAP INNO-BIA AlzBio3 immunoassay (Fujirebio, Ghent Belgium) for total tau, p-tau181 and beta-amyloid 1–42 (Aβ42) at the UPenn Biomarker Research Laboratory) or with commercially available ELISA kits (Covance laboratory, Dedham, MA) as described in detail elsewhere[22].
3. Structural MRI with minimum requirements for these analyses were obtained at baseline and annually, and were available for a subset of participants at baseline (N = 160). These participants were enrolled at 10 PPMI sites that used a standardized protocol for 3 Tesla machines (all Siemens Healthcare, USA). A 3D magnetization prepared rapid gradient echo (MPRAGE) sequence was used for imaging brain anatomy (176 axial slices, repetition time = 2300 ms, echo time = 2.98 ms, flip angle = 9°, voxel size 1 × 1 × 1 mm^3^). The images were centrally processed at UCSF for cortical and subcortical morphometric measurements using FreeSurfer version 5.1[23]. FreeSurfer is a suite of algorithmic tools that automatically creates models of most anatomical brain structures on MRI based on a subject-independent probabilistic brain atlas in combination with nonlinear image registration of individual images to obtain subject-specific measurements. FreeSurfer version 5.1 uses a longitudinal workflow that estimates brain morphometry unbiased toward the chronological scan order by building first a template image from all time points as an unbiased prior distribution for each subject before computing morphometric deformations for every time point. This strategy reduces the random variation in the processing procedure and improves the robustness and sensitivity of the overall longitudinal analysis. A previous test-retest study validated that the longitudinal processing provides consistent brain parcellation[24]. All raw images as well as the results of brain parcellation underwent a visual quality control by trained technicians. A partial failure rating for gross parcellation errors in 1 or more specific brain regions occurred in about 15% of the image, but none had a complete parcellation failure. The errors also did not appear to be systematic and MRIs with a partial failure rating were still included in the analyses but only the correctly parcellated brain regions were assessed. The outcome measures of the FreeSurfer workflow were 93 automatically-labeled brain regions, including gyri and subcortical structures, for each subject. MRI data for baseline, year 1 and year 2 visits were utilized for volume and thickness for 34 regions, with left and right hemisphere values averaged.
4. Diffusion tensor imaging (DTI) MRI results were available for a subset of participants who also had structural MRI (N = 151). DTI data from 9 of 160 with MRI had to be excluded because of poor data quality. DTI measures were mean diffusivity (MD) and fractional anisotropy (FA) for 61 brain regions, with left and right hemisphere values averaged. A cardiac-gated two-dimensional single-shot echo-planar sequence for mapping brain water DTI (TR ranged from 8,400 to 8,800 depending on subjects’ heart rate, TE = 88ms, 2 mm isotropic resolution; 72 contiguous slices, twofold acceleration, axial-oblique aligned along the anterior-posterior commissure, with diffusion-weighted gradients along 64 sensitization directions and a b factor of 1000s/mm2) was acquired for each participant. Processing images were first visually inspected for significant image artifacts and then processed using an automated processing script designed for longitudinal data analysis. The initial steps include corrections for head motion, eddy-current effects and susceptibility distortions of DTI[25], followed by the computation of standard scalar parameter maps of the diffusion tensor, such as fractional anisotropy (FA), radial diffusivity (rD), and axial diffusivity (aD). An intra-subject affine registration was performed between the parametric DTI maps and the structural T1- and T2- weighted images at baseline. An inter-subject registration was performed for group analysis using the standard protocol of DARTEL, which involves tissue segmentation of the structural images for DARTEL initialization, a diffeomorphic algorithm for inter-subject image registration, and finally a spatial normalization of the registered images to MNI space[26], allowing the anatomical parcellation of the brain according to the JHU-DTI-MNI (Type I WMPM)[27]. To reduce any group bias in the anatomical parcellation, a group-averaged template was created from all subject images in MNI space, followed by a non-linear registration between the JHU-DTI-MNI atlas and the group-averaged template. The JHU-DTI-MNI atlas is reversely transformed to each subject space, facilitating regions-of-interest (ROIs) extraction from each parametric DTI map at baseline. For group analysis, DTI measures were extracted from 118 ROIs in the entire white matter and subcortical regions, including the basal ganglia and brain stem sub-regions. The outcome measures of the FreeSurfer workflow were 93 automatically-labeled brain regions, including gyri and subcortical structures, for each subject, based on the Desikan-Killiany brain structure atlas[28].
5. Genotyping was performed with NeuroX, a genotyping platform comprised of standard Illumina exome content (~240,000 variants) and over 24,000 custom content variants focusing on neurologic diseases (~24,000 variants)[29]. Single nucleotide polymorphisms (SNPs) previously associated with cognitive impairment or decline in PD were examined (i.e., apolipoprotein E4 [*ApoE4*], glucocerebrosidase [*GBA*; N3705], leucine-rich repeat kinase 2 [*LRKK2*; G20195], synuclein [rs3910105 and rs356181], microtubule associated protein tau [*MAPT*; rs17649553, which is in linkage dysequilibrium with the H1 haplotype], brain-derived neurotrophic factor val^66^met [*BDNF* val^66^met], and Catechol-O-methyltransferase val^158^met [*COMT* val^158^met]). rs17649553 is in strong linkage dysequilibrium with an “H1 tagging” SNP (rs242928; D’ = 0.991, R2 = 0.203).

#### Statistical analysis

Longitudinal logistic or linear mixed-effect models were used to find baseline and longitudinal predictors (treated as time-dependent predictors) of cognitive impairment over the 3-year time period. The following covariates were considered for each cognitive outcome: age, sex, race, education level, levodopa equivalent daily dose (LEDD)[30], baseline MDS Unified Parkinson’s Disease Rating Scale (MDS-UPDRS) motor score, baseline depression (GDS-15 score ≥5), baseline psychosis (MDS-UPDRS 1.2 item score >0), and baseline REM sleep behavior disorder (RBDSQ score ≥5). To select the most appropriate set of covariates for each outcome, a combination of Akaike information criteria (AIC) fit statistics and univariate p-values were used to perform a backwards selection of covariates to find the best model fit. AIC fit statistics were also used to determine whether site should be included as a random effect for each outcome. In addition to these selected covariates, models examining MRI volume also adjusted for total intracranial volume (ICV), and models examining MRI DTI measures also adjusted for white matter density in each individual region examined.

After covariates and random effects were selected for each outcome, univariate analyses were run for each biomarker variable to predict cognitive impairment over time. Due to the large number of predictors, a false discovery rate (FDR) approach (FDR = 0.2) was used to select biomarker variables from the univariate analyses for inclusion in multivariable models run with other biomarkers. Then, variables were removed from the multivariable model individually in a backwards selection process until all remaining variables were significant at 0.1 level. To avoid collinearity with biomarkers, the following rules were used when fitting the multivariable models: if contralateral putamen or caudate measures were significant in a univariate manner, they were considered in the multivariable model. If not, but ipsilateral putamen or caudate measures were significant in a univariate manner, they were considered in the multivariable model. Similarly, if any of the individual CSF biomarkers were significant in a univariate manner, they were considered in the multivariable model; CSF ratios were only considered in the multivariable model if neither of the individual biomarkers was significant.

As structural and diffusion tensor MRIs were only available in a subset of patients, two populations were analyzed for each cognitive outcome: (1) the subset of participants with MRI data (these models included MRI plus other biomarker data), and (2) the full population (these models included only other biomarker data). Separate models were run for baseline predictors (all biomarkers) and longitudinal predictors (DAT imaging and structural MRI).

All statistical analyses were performed using SAS 9.4 (SAS Institute Inc., Cary, NC).

## Results

### Participant characteristics

Baseline demographic and clinical characteristics for all PD participants (N = 423) are in Table 1. The cohort is approximately two-thirds male, overwhelmingly white, and highly educated. The characteristics for the subset of participants with MRI data (N = 160) was similar to that of the full population. Table 2 lists the number of PD participants with biomarker availability at each time point. Genetic, CSF, and DTI testing was done at baseline, and DAT imaging and MRI thickness and volumes at baseline, year 1 and year 2.

**Table 1:** Baseline demographic and clinical characteristics.

Variable	All PD participants	PD participants with MRI data
	(N = 423)	(N = 160)
**Age**		
Mean years (SD; minimum, maximum)	61.7 (9.7; 33.5, 84.9)	61.0 (9.6; 38.0, 82.3)
**Gender**		
Male	277 (65.5%)	103 (64.4%)
Female	146 (34.5%)	57 (35.6%)
**Education**		
<13 years	76 (18.0%)	38 (23.8%)
13–23 years	344 (81.3%)	122 (76.3%)
>23 years	3 (0.7%)	0 (0.0%)
**Race**		
White	391 (92.4%)	151 (94.4%)
Black/African-American	6 (1.4%)	3 (1.9%)
Asian	8 (1.9%)	3 (1.9%)
Other	18 (4.3%)	3 (1.9%)
**Duration of disease (months)**		
Mean (SD; minimum, maximum)	6.7 (6.5, 0.4, 35.8)	6.87 (7.0; 0.4, 35.8)
**MDS-UPDRS Part III score**		
Mean (SD; minimum, maximum)	20.9 (8.9; 4.0, 51.0)	20.9 (9.1; 4.0, 47.0)
**GDS score (score ≥5)**	59 (13.9%)	25 (15.6%)
**MDS-UPDRS psychosis (score ≥1)**	13 (3.1%)	6 (3.8%)

**Table 2:** Biomarker availability at baseline and longitudinally.

Biomarker	Number PD participants			
	Baseline	Year 1	Year 2	Year 3
Genotyping	384	n/a	n/a	n/a
DTI FA and MD	151	n/a	n/a	n/a
MRI volume and thickness	160	148	110	n/a
CSF	412	n/a	n/a	n/a
DAT scan	418	358	296	n/a

### Cognitive outcomes over time

Cognitive assessments were available for up to 423 participants at baseline, 395 at year 1, 376 at year 2, and 239 at year 3 (dropout rate <10%, so the decreasing number of participants over time is largely due to the fact that many participants had not yet reached year 3 of study participation at the time of data download).

Over the 3-year period the mean MoCA score declined by approximately 1 point on average, and the frequency of participants screening positive for cognitive impairment (i.e., MoCA score <26) increased from 22% to 37%, with dementia-level impairment (i.e., MoCA score <21[20]) increasing from 1% to 6% over time, see Table 3. Cognitive impairment increased from 11% to 15% based on detailed neuropsychological test results. Using the site investigator’s diagnostic determination, the diagnosis of MCI increased from 9% to 21% and PDD from 0% to 3%.

**Table 3:** Cognitive outcomes over time.

Variable	PD Subjects				Change from Baseline to Year 3 (p value)
	Baseline	Year 1	Year 2	Year 3	
	(N = 423)	(N = 395)	(N = 376)	(N = 239)	
MoCA score					<0.001
N	423	392	371	238	
Mean (SD)	27.13 (2.3)	26.30 (2.8)	26.26 (3.2)	26.02 (3.3)	
(Min, Max)	(17.0, 30.0)	(15.0, 30.0)	(9.0, 30.0)	(13.0, 30.0)	
MoCA score <26					<0.001
N	423	392	371	238	
Yes	93 (22.0%)	135 (34.4%)	121 (32.6%)	89 (37.4%)	
MoCA score <21					0.002
N	423	392	371	236	
Yes	4 (0.9%)	13 (3.3%)	20 (5.4%)	13 (5.5%)	
JLO score					0.02
N	422	394	369	236	
Mean (SD)	12.77 (2.1)	12.33 (2.4)	12.82 (2.3)	12.56 (2.4)	
(Min, Max)	(5.0, 15.0)	(2.0, 15.0)	(0.0, 15.0)	(3.0, 15.0)	
HVLT immediate recall score					0.54
N	422	394	374	238	
Mean (SD)	24.44 (5.0)	23.82 (5.4)	23.71 (5.5)	24.19 (6.1)	
(Min, Max)	(9.0, 36.0)	(4.0, 36.0)	(9.0, 36.0)	(6.0, 36.0)	
HVLT-R delayed recall score					0.06
N	422	394	374	237	
Mean (SD)	8.36 (2.5)	8.10 (2.9)	8.21 (3.0)	8.08 (3.0)	
(Min, Max)	(0.0, 12.0)	(0.0, 12.0)	(0.0, 12.0)	(0.0, 12.0)	
HVLT-R retention score					0.38
N	421	392	374	236	
Mean (SD)	11.18 (1.2)	11.14 (1.4)	11.26 (1.7)	11.08 (1.6)	
(Min, Max)	(0.0, 12.0)	(0.0, 12.0)	(0.0, 12.0)	(0.0, 12.0)	
HVLT-R discrimination recognition score					0.69
N	421	392	374	236	
Mean (SD)	9.63 (2.6)	9.67 (2.5)	10.68 (2.4)	9.69 (2.5)	
(Min, Max)	(-4.0, 12.0)	(-1.0, 12.0)	(-2.0, 12.0)	(-2.0, 12.0)	
LNS score					0.006
N	422	393	374	238	
Mean (SD)	10.59 (2.7)	10.36 (2.7)	10.32 (2.8)	10.15 (3.0)	
(Min, Max)	(2.0, 20.0)	(2.0, 18.0)	(2.0, 19.0)	(1.0, 18.0)	
Semantic fluency total score					0.04
N	422	393	374	238	
Mean (SD)	48.67 (11.6)	48.75 (11.5)	48.98 (13.0)	47.47 (11.3)	
(Min, Max)	(20.0, 103.0)	(18.0, 97.0)	(15.0, 95.0)	(9.0, 86.0)	
SDMT score					<0.001
N	422	394	373	236	
Mean (SD)	41.18 (9.7)	40.78 (10.3)	39.95 (11.1)	39.14 (11.7)	
(Min, Max)	(7.0, 82.0)	(5.0, 70.0)	(2.0, 75.0)	(0.0, 65.0)	
2 scores >1.5 SD below standardized mean					0.05
N	415	386	360	226	
Yes	44 (10.6%)	52 (13.5%)	45 (12.5%)	33 (14.6%)	
Site investigator diagnosis cognitive impairment					0.001
N	106	271	366	235	
Normal	97 (91.5%)	231 (85.2%)	306 (83.6%)	179 (76.2%)	
Mild cognitive impairment	9 (8.5%)	38 (14.0%)	57 (15.6%)	50 (21.3%)	
Dementia	0 (0.0%)	2 (0.7%)	3 (0.8%)	6 (2.6%)	

MoCA = Montreal Cognitive Assessment.

JLO = Benton Judgment of Line Orientation.

HVLT-R = Hopkins Verbal Learning Test-Revised.

LNS = Letter-Number Sequencing.

SDMT-Symbol-Digit Modalities Test.

### Neurobiological predictors of cognitive impairment

#### Global cognitive impairment (MoCA)

Baseline CSF, DAT imaging, DTI (MD and FA), and MRI (volume and thickness) values, and the eight SNPs examined, did not predict MoCA score <26 over time (data not shown). In the subset of patients with MRI data, baseline decreased entorhinal (p = 0.007) and superior temporal lobe (p = 0.004) volumes were associated with greater decline in MoCA score over time.

Longitudinal DAT imaging did not predict MoCA score <26 over time. In the subset of patients with MRI data, decreased caudal middle frontal (p = 0.096), superior parietal (p = 0.03), and superior temporal (p = 0.08) volumes over time were associated with MoCA score <26 over time. Decreased lateral orbitofrontal (p = 0.05), superior parietal (p = 0.007), and superior temporal (p = 0.07) volumes, and decreased precentral thickness (p = 0.02), over time predicted greater decline in continuous MoCA score over time.

#### Neuropsychological test-defined cognitive impairment

Baseline CSF, DAT imaging, DTI (MD and FA), and MRI (volume and thickness) values, and the eight SNPs examined, did not predict test-based cognitive impairment over time (data not shown). Likewise, longitudinal DAT imaging and MRI (volume and thickness) values did not predict test-based cognitive impairment over time (data not shown).

#### Site investigator diagnosis of cognitive impairment

Table 4 shows lower baseline ipsilateral caudate DAT availability and CSF Aβ 1–42 predicted cognitive impairment after FDR correction and on multivariable analysis. Smaller fusiform, lateral occipital, and lateral orbitofrontal (for MRI volume) and decreased inferior cerebellar peduncle MD (for DTI MD) predicted cognitive impairment in multivariable analyses, see S1 Table.

**Table 4:** Baseline biomarker predictors of investigator diagnosis of cognitive impairment.

Variable	PD Subjects (N = 403)			
	Univariate p-value	# Subjects	Multivariable Analysis	
		Missing	OR (95% CI)	p-value
**CSF Biologics**				
Alpha-Synuclein	0.87	13	-	-
A-Beta 1–42	<0.001	13	0.995 (0.992, 0.998)	0.001
t-tau	0.87	17	-	-
p-tau	0.56	15	-	-
t-tau/A-Beta 1–42	0.02	17	Not included	NA
p-tau/A-Beta 1–42	0.13	15	-	-
p-tau/t-tau	0.51	19	-	-
**Genetics**				
*ApoE4*	0.67	40	-	-
*GBA* N370S	0.18	37	-	-
*LRRK2* G2019S	0.31	36	-	-
*MAPT* rs17649553	0.11	36	-	-
*SNCA* rs3910105	0.36	36	-	-
*SNCA* rs356181	0.96	36	-	-
*BDNF* val66met	0.09	36	-	-
*COMT* val158met	0.05	36	-	-
**DAT imaging**				
Contralateral Caudate	0.09	6	-	-
Ipsilateral Caudate	0.03	6	0.450 (0.237, 0.855)	0.01
Contralateral Putamen	0.48	6	-	-
Ipsilateral Putamen	0.24	6	-	-

Table 5 shows lower contralateral caudate DAT availability over time was associated with cognitive impairment in multivariable analyses. In addition, smaller fusiform and superior temporal lobe volumes, and larger caudal anterior cingulate and smaller fusiform thickness, over time were associated with cognitive impairment in multivariate analyses, see S2 Table.

**Table 5:** Longitudinal biomarker predictors of investigator diagnosis of cognitive impairment.

Variable	PD Subjects (N = 365)			
	Univariate p-value	# Subjects	Multivariable Analysis	
		Missing	OR (95% CI)	p-value
**DAT imaging**				
Contralateral Caudate	0.05	2	0.484 (0.237, 0.989)	0.05
Ipsilateral Caudate	0.03	2	Not included	NA
Contralateral Putamen	0.22	2	-	-
Ipsilateral Putamen	0.16	2	-	-

Examining the entire cohort (i.e., excluding MRI variables) and including only patients who were cognitively intact at baseline (N = 394), baseline predictors of incident cognitive impairment (based on site investigator diagnosis) were lower CSF Aβ 1–42, lower ipsilateral caudate DAT availability, *COMT* val158met (val/val genotype), and *BDNF* val66met (val/val genotype), see S3 Table. A longitudinal biomarker predictor of incident cognitive impairment was decreased contralateral putamen DAT availability (p = 0.07).

## Discussion

In this multi-modal longitudinal examination of predictors of cognitive impairment in early PD, the biomarkers in general predicting cognitive impairment that remained significant in multivariable models were: (1) dopamine deficiency; (2) brain-wide decreased volume or thickness; (3) white matter tract abnormalities; (4) possible co-morbid AD pathology; and (5) genetic SNPs summarized in S4 Table.

By year three after PD diagnosis, cognitive impairment was diagnosed in 15–37% participants and increased in frequency by 50–200% over this time period depending on the criteria applied, consistent with the relatively high frequency[4, 31] and worsening over time[32] reported in other early PD cohorts.

There were no biological predictors of neuropsychological test-defined impairment; one possible explanation is that the smallest percentage of participants fulfilled this criterion for cognitive impairment over time (15% versus either 24% or 37% for the other criteria). The greatest evidence for biomarkers predicting cognitive decline in this early, relatively cognitively intact population occurred when using the site investigator’s annual diagnosis of cognitive impairment.

We found that both caudate and putamen DAT deficits, either at disease onset or worsening over time, predicted cognitive impairment. This confirms previous cross-sectional and longitudinal research in early PD using DAT[33] or other striatal dopamine system imaging ligands[34]. These findings suggest that enhancing dopamine function in early PD might improve cognitive abilities, at least acutely or temporarily[35], and that serial DAT imaging might serve as a cognitive biomarker in PD cognition studies.

Lower CSF Aβ 1–42 levels, suggestive of co-morbid AD Aβ amyloid brain deposition, have been associated with memory impairment in de novo PD patients[31] and as well as future cognitive decline[36, 37]. AD pathology is associated with increasing age in PD, but here an association was shown between cognitive impairment and baseline Aβ 1–42 levels, when the mean age of patients was only 62 years, suggesting that AD-related changes in PD can occur at a relatively young age and long prior to the development of dementia, as reported for MCI in the general population[38] and in preliminary PD neuropathological studies[39].

Multiple, widely spread brain regions of decreased volume, and to a lesser extent thickness, predicted cognitive impairment, and for some brain regions cognitive impairment was predicted by ongoing atrophy, including frontal, parietal, temporal and occipital lobe regions. These findings overlap with previous findings of temporal-parietal and frontal atrophy and thinning with MCI in early PD [40]. It is possible that the cortical atrophy observed in vivo using structural MRI is associated with cortical PD- or AD-related neuropathological changes, which would be consistent with neuropathology studies showing that both cortical LBD pathology and co-morbid Aβ amyloid plaque deposits are associated with cognitive impairment in PD[41].

Specific brain regions (associated cognitive function) implicated included the lateral occipital (object recognition and spatial vision), lateral orbitofrontal (executive abilities), and entorhinal (memory) cortices, subserving cognitive abilities that can be impaired early in the course of PD. The latter finding is consistent with recent research that medial temporal lobe atrophy is associated with cognitive impairment and decline in non-demented PD patients[42].

Neither increased MD nor decreased FA predicted cognitive impairment. Previous research in de novo PD reported an association between increased MD in frontal and parietal white matter tracts and specific cognitive tests[43]. Cohort and study design differences may in part explain these discrepant findings, but the analyses performed here were more stringent than those utilized in previous research.

Two SNPs associated with cognitive decline, the *COMT* val158met SNP and *BDNF* val66met. There is a complex association between the *COMT* val158met SNP and cognition in PD, influenced by both disease severity and use of dopaminergic medication[44]. In our analyses, the high activity *COMT* val158met genotype was associated with cognitive impairment. Regarding *BDNF*, its product is important for survival and differentiation of dopaminergic neurons in the basal ganglia. A recent study found that the *BDNF* val-allele carriers had great decline in executive abilities over time, consistent with our findings [45].

Unlike some previous studies, we did not show an association between *ApoE4* or *MAPT* status and cognitive impairment. For *ApoE4*, it is important to note that most previous studies have focused on PD patients with dementia[46], and the PPMI sample is relatively young and cognitively intact. For *MAPT*, the H1 haplotype has been associated with cognitive decline or dementia in some[47] but not all[11] PD studies. Longer duration of follow up of this cohort will unveil if genetic risks are important in later-onset or more advanced cognitive dysfunction.

Strengths of the study are inclusion of multiple and international sites; the relatively large sample size; inclusion of multiple biomarkers, including some obtained serially; enrollment of participants starting at symptom onset; annual cognitive and clinical assessments; use of four definitions of cognitive impairment; and a stringent, multi-step statistical analysis plan. Limitations include: highly educated and overwhelmingly white cohort limiting generalizability; variable sample sizes for the different biomarkers, with less than half the patients having research quality MRI scans for inclusion; although CSF is collected serially in PPMI, currently only baseline values are available; the cognitive battery utilized in PPMI is limited and the site investigator’s diagnosis of cognitive impairment was available for the entire cohort only starting at year two; other biomarkers associated with early cognitive decline in PD (e.g., measures of cholinergic integrity and FDG-PET) are not included in PPMI; and lack of comparison with the healthy controls enrolled in PPMI to assess the disease specificity of our findings.

Cognitive decline in early PD is independently predicted by multiple biomarker changes, including nigrostriatal dopamine system deficits, wide-ranging atrophy consistent with cortical neurodegenerative disease, evidence for co-morbid AD pathology, and genetic factors. This provides confirmation for heterogeneity in the neural substrate of the early cognitive deficits in PD, and highlights the need to incorporate multiple biomarkers when risk factors for cognitive decline. Validation and extension of these findings will help in the design of clinical trials for cognitive impairment in PD, including those testing possible disease-modifying therapies from disease onset, and also be a step toward personalized medicine.

## Supporting information

S1 Table Baseline biomarker predictors of investigator diagnosis of cognitive impairment in participants with MRI data. (DOCX)

S2 Table Longitudinal biomarker predictors of investigator diagnosis of cognitive impairment in participants with MRI data. (DOCX)

S3 Table Baseline biomarker predictors of incident cognitive impairment. (DOCX)

S4 Table Summary table of significant biomarker predictors of cognitive impairment. (DOCX)
