# Supplementary material for: Multiple modality biomarker prediction of cognitive impairment in prospectively followed de novo Parkinson disease
Source: PLoS One. 2017 May 17;12(5):e0175674. doi: 10.1371/journal.pone.0175674 (PMC5435130; doi:10.1371/journal.pone.0175674)
Supplement: S1 Table — (DOCX) [file pone.0175674.s001.docx]

**Table A in S1 File. Baseline biomarker predictors of investigator diagnosis of cognitive impairment in participants with MRI data**

| **Variable** | **PD Subjects (N = 154)** | | | |
| --- | --- | --- | --- | --- |
|  | **Univariate**  **p-value** | **# Subjects** | **Multivariable Analysis** | |
|  |  | **Missing** | **OR (95% CI)** | **p-value** |
| **CSF Biologics** |  |  |  | |
| Alpha-Synuclein | 0.18 | 4 | - | - |
| A-Beta 1-42 | 0.15 | 4 | - | - |
| t-tau | 0.13 | 6 | - | - |
| p-tau | 0.71 | 6 | - | - |
| t-tau/A-Beta 1-42 | 0.05 | 6 | - | - |
| p-tau/A-Beta 1-42 | 0.52 | 6 | - | - |
| p-tau/t-tau | 0.33 | 8 | - | - |
| **Genetics** |  |  |  | |
| *ApoE4* | 0.77 | 19 | - | - |
| *GBA* N370S | 0.65 | 12 | - | - |
| *MAPT* rs17649553 | 0.12 | 11 | - | - |
| *SNCA* rs3910105 | 0.09 | 11 | - | - |
| *SNCA* rs356181 | 0.39 | 11 | - | - |
| *BDNF* val66met | 0.15 | 11 | - | - |
| *COMT* val158met | 0.15 | 11 | - | - |
| **DAT imaging** |  |  |  | |
| Contralateral Caudate | 0.95 | 3 | - | - |
| Ipsilateral Caudate | 0.90 | 3 | - | - |
| Contralateral Putamen | 0.07 | 3 | - | - |
| Ipsilateral Putamen | 0.34 | 3 | - | - |
| **MRI Volume** |  |  |  | |
| Banks Superior Temporal Sulcus | 0.004 | 4 | NS | NS |
| Caudal Anterior Orbital | 0.35 | 4 | - | - |
| Caudal Middle Frontal | 0.003 | 9 | NS | NS |
| Cuneus | 0.20 | 7 | - | - |
| Entorhinal | 0.59 | 4 | - | - |
| Fusiform | <0.001 | 5 | 0.502 (0.240, 1.048) | 0.07 |
| Inferior Parietal | <0.001 | 6 | NS | NS |
| Inferior Temporal | 0.006 | 5 | NS | NS |
| Isthmus Cingulate | 0.74 | 4 | - | - |
| Lateral Occipital | <0.001 | 7 | 0.558 (0.304, 1.022) | 0.06 |
| Lateral Orbitofrontal | <0.001 | 4 | 0.234 (0.057, 0.970) | 0.05 |
| Lingual | 0.12 | 7 | - | - |
| Medial Orbitofrontal | 0.24 | 9 | - | - |
| Middle Temporal | 0.002 | 5 | NS | NS |
| Parahippocampal | 0.54 | 4 | - | - |
| Paracentral | 0.28 | 6 | - | - |
| Pars Opercularis | 0.02 | 4 | - | - |
| Pars Orbitalis | 0.01 | 4 | NS | NS |
| Pars Triangularis | 0.47 | 4 | - | - |
| Pericalcarine | 0.40 | 7 | - | - |
| Postcentral | 0.01 | 6 | NS | NS |
| Posterior Cingulate | 0.60 | 4 | - | - |
| Precentral | 0.16 | 9 | - | - |
| Precuneus | 0.02 | 4 | - | - |
| Rostral Anterior Cingulate | 0.09 | 4 | - | - |
| Rostral Middle Frontal | 0.002 | 9 | NS | NS |
| Superior Frontal | 0.274 | 9 | - | - |
| Superior Parietal | 0.287 | 6 | - | - |
| Superior Temporal | <0.001 | 5 | NS | NS |
| Supramarginal | 0.009 | 6 | NS | NS |
| Frontal Pole | 0.74 | 9 | - | - |
| Temporal Pole | 0.69 | 5 | - | - |
| Transverse Temporal | 0.21 | 4 | - | - |
| Insula | 0.10 | 4 | - | - |
| **MRI Thickness** |  |  |  | |
| Banks Superior Temporal Sulcus | 0.42 | 0 | - | - |
| Caudal Anterior Cingulate | 0.03 | 0 | - | - |
| Caudal Middle Frontal | 0.42 | 5 | - | - |
| Cuneus | 0.79 | 3 | - | - |
| Entorhinal | 0.33 | 0 | - | - |
| Fusiform | 0.03 | 1 | - | - |
| Inferior Parietal | 0.15 | 2 | - | - |
| Inferior Temporal | 0.06 | 1 | - | - |
| Isthmus Cingulate | 0.79 | 0 | - | - |
| Lateral Occipital | 0.52 | 3 | - | - |
| Lateral Orbitofrontal | 0.80 | 0 | - | - |
| Lingual | 0.76 | 3 | - | - |
| Medial Orbitofrontal | 0.63 | 5 | - | - |
| Middle Temporal | 0.41 | 1 | - | - |
| Parahippocampal | 0.74 | 0 | - | - |
| Paracentral | 0.73 | 2 | - | - |
| Pars Opercularis | 0.79 | 0 | - | - |
| Pars Orbitalis | 0.80 | 0 | - | - |
| Pars Triangularis | 0.24 | 0 | - | - |
| Pericalcarine | 0.08 | 3 | - | - |
| Postcentral | 0.28 | 2 | - | - |
| Posterior Cingulate | 0.48 | 0 | - | - |
| Precentral | 0.78 | 5 | - | - |
| Precuneus | 0.74 | 0 | - | - |
| Rostral Anterior Cingulate | 0.39 | 0 | - | - |
| Rostral Middle Frontal | 0.53 | 5 | - | - |
| Superior Frontal | 0.41 | 5 | - | - |
| Superior Parietal | 0.37 | 2 | - | - |
| Superior Temporal | 0.59 | 1 | - | - |
| Supramarginal | 0.37 | 2 | - | - |
| Frontal Pole | 0.56 | 5 | - | - |
| Temporal Pole | 0.72 | 1 | - | - |
| Transverse Temporal | 0.94 | 0 | - | - |
| Insula | 0.35 | 0 | - | - |
| **MRI DTI Fractional Anisotropy** |  |  |  | |
| Thalamus | 0.92 | 9 | - | - |
| Caudate Nucleus | 0.17 | 9 | - | - |
| Putamen | 0.97 | 9 | - | - |
| Globus Pallidus | 0.24 | 9 | - | - |
| Substantia Nigra | 0.93 | 9 | - | - |
| Red Nucleus | 0.43 | 9 | - | - |
| Midbrain | 0.43 | 9 | - | - |
| Pons | 0.27 | 9 | - | - |
| Subthalamic Nucleus | 0.98 | 9 | - | - |
| Anterior corona radiata | 0.62 | 9 | - | - |
| Anterior limb of internal capsule | 0.79 | 9 | - | - |
| Cingulum (Rostral anterior) | 0.74 | 9 | - | - |
| Cingulum (Posterior) | 0.46 | 9 | - | - |
| Cingulum (Isthmus) | 0.56 | 9 | - | - |
| Cingulum (Caudal anterior) | 0.34 | 9 | - | - |
| Cingulum (parahippocampus) | 0.97 | 9 | - | - |
| External capsule | 0.33 | 9 | - | - |
| Fornix (cres) / Stria terminalis | 0.09 | 9 | - | - |
| Inferior fronto-occipital fasciculus | 0.92 | 9 | - | - |
| Posterior corona radiata | 0.88 | 9 | - | - |
| Posterior limb of internal capsule | 0.50 | 9 | - | - |
| Posterior thalamic radiation (include optic radiation) | 0.38 | 9 | - | - |
| Retrolenticular part of internal capsule | 0.70 | 9 | - | - |
| Superior corona radiata | 0.08 | 9 | - | - |
| Superior fronto-occipital fasciculus | 0.92 | 9 | - | - |
| Superior longitudinal fasciculus | 0.69 | 9 | - | - |
| Sagittal stratum/inferior longitudinal fasciculus | 0.09 | 9 | - | - |
| Uncinate fasciculus | 0.74 | 9 | - | - |
| Cerebral peduncle | 0.33 | 9 | - | - |
| Corticospinal tract | 0.98 | 9 | - | - |
| Inferior cerebellar peduncle | 0.08 | 9 | - | - |
| Middle cerebellar peduncle | 0.28 | 9 | - | - |
| Superior cerebellar peduncle | 0.90 | 9 | - | - |
| Medial lemniscus | 0.42 | 9 | - | - |
| Pontine crossing tract (a part of MCP) | 0.31 | 9 | - | - |
| Tapatum | 0.73 | 9 | - | - |
| Rectus WM | 0.59 | 9 | - | - |
| Middle fronto-orbital WM | 0.15 | 9 | - | - |
| Lateral fronto-orbital WM | 0.11 | 9 | - | - |
| Inferior frontal WM | 0.15 | 9 | - | - |
| Middle frontal WM | 0.18 | 9 | - | - |
| Superior frontal WM | 0.17 | 9 | - | - |
| Precentral WM | 0.15 | 9 | - | - |
| Postcentral WM | 0.40 | 9 | - | - |
| Superior parietal WM | 0.29 | 9 | - | - |
| Supramarginal WM | 0.51 | 9 | - | - |
| Angular WM | 0.14 | 9 | - | - |
| Precuneus WM | 0.85 | 9 | - | - |
| Superior occipital WM | 0.13 | 9 | - | - |
| Middle occipital WM | 0.03 | 9 | - | - |
| Inferior occipital WM | 0.08 | 9 | - | - |
| Cuneus WM | 0.69 | 9 | - | - |
| Lingual WM | 0.37 | 9 | - | - |
| Fusiform WM | 0.38 | 9 | - | - |
| Superior temporal WM | 0.28 | 9 | - | - |
| Middle temporal WM | 0.68 | 9 | - | - |
| Inferior temporal WM | 0.45 | 9 | - | - |
| Genu of corpus callosum | 0.97 | 9 | - | - |
| Body of corpus callosum | 0.92 | 9 | - | - |
| Splenium of corpus callosum | 0.59 | 9 | - | - |
| Fornix | 0.55 | 9 | - | - |
| **MRI DTI Mean Diffusivity** |  |  |  |  |
| Thalamus | 0.09 | 9 | - | - |
| Caudate Nucleus | 0.17 | 9 | - | - |
| Putamen | 0.49 | 9 | - | - |
| Globus Pallidus | 0.47 | 9 | - | - |
| Substantia Nigra | 0.76 | 9 | - | - |
| Red Nucleus | 0.25 | 9 | - | - |
| Midbrain | 0.91 | 9 | - | - |
| Pons | 0.63 | 9 | - | - |
| Subthalamic Nucleus | 0.44 | 9 | - | - |
| Anterior corona radiata | 0.94 | 9 | - | - |
| Anterior limb of internal capsule | 0.91 | 9 | - | - |
| Cingulum (Rostral anterior) | 0.19 | 9 | - | - |
| Cingulum (Posterior) | 0.09 | 9 | - | - |
| Cingulum (Isthmus) | 0.39 | 9 | - | - |
| Cingulum (Caudal anterior) | 0.54 | 9 | - | - |
| Cingulum (parahippocampus) | 0.84 | 9 | - | - |
| External capsule | 0.04 | 9 | - | - |
| Fornix (cres) / Stria terminalis | 0.10 | 9 | - | - |
| Inferior fronto-occipital fasciculus | 0.43 | 9 | - | - |
| Posterior corona radiata | 0.32 | 9 | - | - |
| Posterior limb of internal capsule | 0.71 | 9 | - | - |
| Posterior thalamic radiation (include optic radiation) | 0.78 | 9 | - | - |
| Retrolenticular part of internal capsule | 0.10 | 9 | - | - |
| Superior corona radiata | 0.84 | 9 | - | - |
| Superior fronto-occipital fasciculus | 0.77 | 9 | - | - |
| Superior longitudinal fasciculus | 0.72 | 9 | - | - |
| Sagittal stratum/inferior longitudinal fasciculus | 0.31 | 9 | - | - |
| Uncinate fasciculus | 0.22 | 9 | - | - |
| Cerebral peduncle | 0.04 | 9 | - | - |
| Corticospinal tract | 0.75 | 9 | - | - |
| Inferior cerebellar peduncle | 0.01 | 9 | 0.113 (0.023, 0.563)* | 0.008 |
| Middle cerebellar peduncle | 0.47 | 9 | - | - |
| Superior cerebellar peduncle | 0.29 | 9 | - | - |
| Medial lemniscus | 0.47 | 9 | - | - |
| Pontine crossing tract (a part of MCP) | 0.56 | 9 | - | - |
| Tapatum | 0.10 | 9 | - | - |
| Rectus WM | 0.91 | 9 | - | - |
| Middle fronto-orbital WM | 0.69 | 9 | - | - |
| Lateral fronto-orbital WM | 0.78 | 9 | - | - |
| Inferior frontal WM | 0.07 | 9 | - | - |
| Middle frontal WM | 0.13 | 9 | - | - |
| Superior frontal WM | 0.60 | 9 | - | - |
| Precentral WM | 0.09 | 9 | - | - |
| Postcentral WM | 0.61 | 9 | - | - |
| Superior parietal WM | 0.17 | 9 | - | - |
| Supramarginal WM | 0.58 | 9 | - | - |
| Angular WM | 0.08 | 9 | - | - |
| Precuneus WM | 0.01 | 9 | NS | NS |
| Superior occipital WM | 0.10 | 9 | - | - |
| Middle occipital WM | 0.08 | 9 | - | - |
| Inferior occipital WM | 0.16 | 9 | - | - |
| Cuneus WM | 0.02 | 9 | - | - |
| Lingual WM | 0.11 | 9 | - | - |
| Fusiform WM | 0.27 | 9 | - | - |
| Superior temporal WM | 0.52 | 9 | - | - |
| Middle temporal WM | 0.68 | 9 | - | - |
| Inferior temporal WM | 0.55 | 9 | - | - |
| Genu of corpus callosum | 0.62 | 9 | - | - |
| Body of corpus callosum | 0.15 | 9 | - | - |
| Splenium of corpus callosum | 0.81 | 9 | - | - |
| Fornix | 0.07 | 9 | - | - |

*OR for 0.1 unit increase.

Note: All analyses adjust for age, gender, race, education level, baseline MDS-UPDRS motor score, baseline psychosis, and baseline RBD, with nested random effects for subjects within sites.
