# Supplementary material for: Multiple modality biomarker prediction of cognitive impairment in prospectively followed de novo Parkinson disease
Source: PLoS One. 2017 May 17;12(5):e0175674. doi: 10.1371/journal.pone.0175674 (PMC5435130; doi:10.1371/journal.pone.0175674)
Supplement: S2 Table — (DOCX) [file pone.0175674.s002.docx]

**Table B in S2 File. Longitudinal biomarker predictors of investigator diagnosis of cognitive impairment in participants with MRI data**

|  | **PD Subjects (N = 154)** | | | |
| --- | --- | --- | --- | --- |
| **Variable** | **Univariate**  **p-value** | **# Subjects** | **Multivariable Analysis** | |
|  |  | **Missing** | **OR (95% CI)** | **p-value** |
| **DaTscan** |  |  |  | |
| Contralateral Caudate | 0.88 | 12 | - | - |
| Ipsilateral Caudate | 0.96 | 12 | - | - |
| Contralateral Putamen | 0.92 | 12 | - | - |
| Ipsilateral Putamen | 0.49 | 12 | - | - |
| **MRI Volume** |  |  |  | |
| Banks Superior Temporal Sulcus | 0.01 | 0 | NS | NS |
| Caudal Anterior Cingulate | 0.19 | 0 | - | - |
| Caudal Middle Frontal | 0.008 | 5 | NS | NS |
| Cuneus | 0.04 | 1 | NS | NS |
| Entorhinal | 0.22 | 0 | - | - |
| Fusiform | <0.001 | 0 | 0.399 (0.193, 0.823) | 0.01 |
| Inferior Parietal | <0.001 | 5 | NS | NS |
| Inferior Temporal | 0.008 | 0 | NS | NS |
| Isthmus Cingulate | 0.53 | 0 | - | - |
| Lateral Occipital | <0.001 | 1 | NS | NS |
| Lateral Orbitofrontal | <0.001 | 0 | NS | NS |
| Lingual | 0.03 | 1 | NS | NS |
| Medial Orbitofrontal | 0.29 | 5 | - | - |
| Middle Temporal | <0.001 | 0 | NS | NS |
| Parahippocampal | 0.35 | 0 | - | - |
| Paracentral | 0.68 | 5 | - | - |
| Pars Opercularis | 0.008 | 0 | NS | NS |
| Pars Orbitalis | 0.007 | 0 | NS | NS |
| Pars Triangularis | 0.31 | 0 | - | - |
| Pericalcarine | 0.22 | 1 | - | - |
| Postcentral | 0.004 | 5 | NS | NS |
| Posterior Cingulate | 0.46 | 0 | - | - |
| Precentral | 0.19 | 5 | - | - |
| Precuneus | 0.006 | 0 | NS | NS |
| Rostral Anterior Cingulate | 0.70 | 0 | - | - |
| Rostral Middle Frontal | 0.09 | 5 | - | - |
| Superior Frontal | 0.005 | 5 | NS | NS |
| Superior Parietal | 0.02 | 5 | NS | NS |
| Superior Temporal | <0.001 | 0 | 0.343 (0.158, 0.747) | 0.008 |
| Supramarginal | 0.005 | 5 | NS | NS |
| Frontal Pole | 0.42 | 5 | - | - |
| Temporal Pole | 0.39 | 0 | - | - |
| Transverse Temporal | 0.11 | 0 | - | - |
| Insula | 0.06 | 0 | - | - |
| **MRI Thickness** |  |  |  | |
| Banks Superior Temporal Sulcus | 0.42 | 0 | - | - |
| Caudal Anterior Cingulate | 0.04 | 0 | 51.864 (0.912, 2951.17) | 0.06 |
| Caudal Middle Frontal | 0.56 | 5 | - | - |
| Cuneus | 0.29 | 1 | - | - |
| Entorhinal | 0.15 | 0 | - | - |
| Fusiform | 0.04 | 0 | 0.522 (0.276, 0.989)* | 0.05 |
| Inferior Parietal | 0.26 | 5 | - | - |
| Inferior Temporal | 0.16 | 0 | - | - |
| Isthmus Cingulate | 0.78 | 0 | - | - |
| Lateral Occipital | 0.73 | 1 | - | - |
| Lateral Orbitofrontal | 0.63 | 0 | - | - |
| Lingual | 0.79 | 1 | - | - |
| Medial Orbitofrontal | 0.87 | 5 | - | - |
| Middle Temporal | 0.28 | 0 | - | - |
| Parahippocampal | 0.25 | 0 | - | - |
| Paracentral | 0.61 | 5 | - | - |
| Pars Opercularis | 0.76 | 0 | - | - |
| Pars fusiform | 0.19 | 0 | - | - |
| Pars Triangularis | 0.52 | 0 | - | - |
| Pericalcarine | 0.80 | 1 | - | - |
| Postcentral | 0.28 | 5 | - | - |
| Posterior Cingulate | 0.52 | 0 | - | - |
| Precentral | 0.76 | 5 | - | - |
| Precuneus | 0.33 | 0 | - | - |
| Rostral Anterior Cingulate | 0.47 | 0 | - | - |
| Rostral Middle Frontal | 0.46 | 5 | - | - |
| Superior Frontal | 0.74 | 5 | - | - |
| Superior Parietal | 0.22 | 5 | - | - |
| Superior Temporal | 0.23 | 0 | - | - |
| Supramarginal | 0.46 | 5 | - | - |
| Frontal Pole | 0.43 | 5 | - | - |
| Temporal Pole | 0.27 | 0 | - | - |
| Transverse Temporal | 0.09 | 0 | - | - |
| Insula | 0.33 | 0 | - | - |
