# Supplementary material for: Multiple modality biomarker prediction of cognitive impairment in prospectively followed de novo Parkinson disease
Source: PLoS One. 2017 May 17;12(5):e0175674. doi: 10.1371/journal.pone.0175674 (PMC5435130; doi:10.1371/journal.pone.0175674)
Supplement: S3 Table — (DOCX) [file pone.0175674.s003.docx]

**Table C in S3 File. Baseline biomarker predictors of incident cognitive impairment**

All analyses adjust for age, gender, race, education level, baseline MDS-UPDRS motor score, baseline psychosis, and baseline RBD, with nested random effects for subjects within sites.
