# Supplementary material for: Multiple modality biomarker prediction of cognitive impairment in prospectively followed de novo Parkinson disease
Source: PLoS One. 2017 May 17;12(5):e0175674. doi: 10.1371/journal.pone.0175674 (PMC5435130; doi:10.1371/journal.pone.0175674)
Supplement: S4 Table — (DOCX) [file pone.0175674.s004.docx]

**Table D in S4 File. Summary table of significant^#^ biomarker predictors of cognitive impairment**

| **Biomarker** | **Definition of cognitive impairment** | | |
| --- | --- | --- | --- |
|  | **MoCA<26 or change in MoCA score** | **Cognitive test deficits (≥2 tests >1.5 SD below mean)** | **Site investigator diagnosis**  **(PD-MCI or PDD)** |
| DAT imaging | - | - | ↓ ipsilateral caudate^*^  ↓ contralateral caudate^§^  ↓ contralateral putamen^§^ |
| CSF | - | - | ↓ CSF Aβ 1-42^*^ |
| Structural MRI (volume) | ↓ entorhinal^*^  ↓ superior temporal^*§^  ↓ caudal middle frontal^§^  ↓ lateral orbitofronal^§^  ↓ superior parietal^§^ | - | ↓ lateral occipital^*^  ↓ lateral orbitofrontal^*^  ↓ fusiform^*§^  ↓ superior temporal^§^ |
| Structural MRI  (thickness) | ↓ precentral^§^ | - | ↑ caudal anterior cingulate^§^  ↓ fusiform^§^ |
| DTI (FA) | - | - | - |
| DTI (MD) | - | - | ↓ inferior cerebellar peduncle^*^ |
| Genetics | - | - | *COMT* val158met (val/val)  *BDNF* val66met (val/val) |

**^#^** Significant in univariate analysis, survived FDR correction, and significant in multivariable analysis

^*^ Baseline biomarker value

^§^ Longitudinal biomarker value
